# Inflammatory Responses in the Secondary Thalamic Injury After Cortical Ischemic Stroke

**DOI:** 10.3389/fneur.2020.00236

**Published:** 2020-04-07

**Authors:** Zhijuan Cao, Sean S. Harvey, Tonya M. Bliss, Michelle Y. Cheng, Gary K. Steinberg

**Affiliations:** ^1^Department of Neurosurgery, Stanford University School of Medicine, Stanford, CA, United States; ^2^Stanford Stroke Center, Stanford University School of Medicine, Stanford, CA, United States

**Keywords:** inflammatory responses, ischemia, secondary injury, stroke, thalamic injury, degeneration

## Abstract

Stroke is one of the major causes of chronic disability worldwide and increasing efforts have focused on studying brain repair and recovery after stroke. Following stroke, the primary injury site can disrupt functional connections in nearby and remotely connected brain regions, resulting in the development of secondary injuries that may impede long-term functional recovery. In particular, secondary degenerative injury occurs in the connected ipsilesional thalamus following a cortical stroke. Although secondary thalamic injury was first described decades ago, the underlying mechanisms still remain unclear. We performed a systematic literature review using the NCBI PubMed database for studies that focused on the secondary thalamic degeneration after cortical ischemic stroke. In this review, we discussed emerging studies that characterized the pathological changes in the secondary degenerative thalamus after stroke; these included excitotoxicity, apoptosis, amyloid beta protein accumulation, blood-brain-barrier breakdown, and inflammatory responses. In particular, we highlighted key findings of the dynamic inflammatory responses in the secondary thalamic injury and discussed the involvement of several cell types in this process. We also discussed studies that investigated the effects of blocking secondary thalamic injury on inflammatory responses and stroke outcome. Targeting secondary injuries after stroke may alleviate network-wide deficits, and ultimately promote stroke recovery.

## Introduction

**Figure 1:**
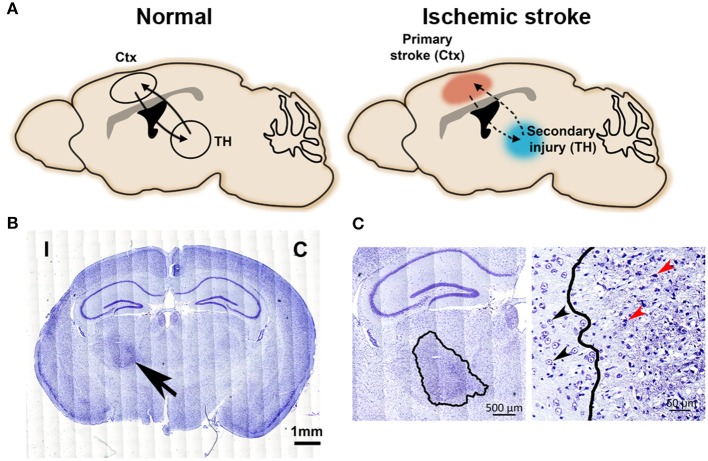
Secondary injury in the connected thalamic nucleus after primary cortical stroke. **(A)** The schematic diagram illustrates neural circuit connections between cortex and thalamus. Left, intact cortico-thalamic and thalamo-cortical circuit connections under normal condition. Right, primary injury in the cortex disrupts cortico-thalamic and thalamo-cortical circuit connections, resulting in secondary injury in the connected thalamus. **(B)** Nissl-stained images show a darkly stained region (arrow) in ipsilesional thalamus at 1-month post-stroke. C, contralesional side; I, ipsilesional side. Scale bar = 1 mm. Adapted from Cao et al. (9), under the CC BY license. **(C)** Left: Enlarged image highlights the degenerative neuronal damage in the ipsilesional thalamus (outlined by a solid line); scale bar = 500 μm. Right: The well-defined boundary between degenerating and healthy neurons in thalamus at 1-month post-stroke. Black arrows indicate normal neurons and red arrows indicate typical injured neurons, scale bar = 50 μm. Adapted from Cao et al. (9), under the CC BY license.

Stroke is a disease with high prevalence and incidence (1). In the USA, ~795,000 people experience a new or recurrent stroke every year (1). Although stroke mortality has decreased in the recent years, a large population of stroke patients still suffers long-term disabilities (1). Increasing efforts have been geared toward understanding brain repair and recovery mechanisms after stroke. In ischemic stroke, the initial cerebral blood flow interruption causes local brain infarct at the acute phase. This damaged primary injury can disrupt network-wide functions, resulting in progressive development of secondary injuries in connected brain regions that can interfere with long-term recovery (2). Thalamus is a key brain region that is particularly affected after cortical stroke (3–5). The secondary injury in the connected ipsilesional thalamus can be detected as early as 3 days after stroke and is still detectable at least after 6 months in rodent and 12 months in patients (6–8). Evidence suggests that this secondary remote injury results in the anterograde/retrograde degeneration after the disruption of functional connections between cortex and thalamus (10, 11) (Figure 1A). The axonal degeneration and dysfunction of the myelin clearance in the distal affected regions also contribute to the secondary lesion development (12). We aimed to present a systematic review of the current understanding of the secondary thalamic injury after ischemic stroke. We summarized the pathological changes associated with secondary thalamic injury and highlighted the findings of the cellular and molecular changes of inflammatory response in the secondary thalamic injury. We also discussed the studies that have investigated the outcome after blocking secondary thalamic injuries in ischemic stroke.

## Literature Search and Review Criteria

**Figure 2:**
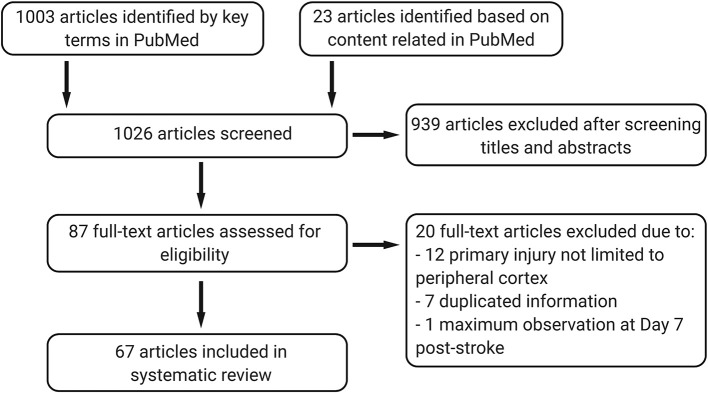
The systematic review progress under Preferred Reporting Items for Systematic Reviews and Meta-Analyses (PRISMA) guidance.

All data are publicly available in the National Center for Biotechnology Information (NCBI) PubMed database. We screened publications in PubMed up to December 31, 2019 for full-text articles in English, using (“ischemic stroke” or “cerebral ischemia” or “middle cerebral artery occlusion” or “middle cerebral artery injury”) AND (“thalamic degeneration” or “thalamic injury” or “thalamic diaschisis” or “secondary degeneration” or “secondary neurodegeneration”). An additional 23 full-text articles from PubMed were also included based on relevant content. We included studies that focused on secondary thalamic degeneration after cortical ischemic stroke. The following studies were excluded: where the primary injury was not limited in cortex, such as global hypoxia stroke or generated by intraluminal suture model or cardiac arrest; duplicate publications; considering the secondary thalamic injury is a delayed injury at chronic stage of stroke, 1 study with the maximum observation time point at Day 7 post-stroke was also excluded. A total of 1,026 articles were screened, and 67 articles were included in this review (Figure 2). No registered review protocol was used in this systematic review. Guidelines from the Preferred Reporting Items for Systematic Reviews and Meta-Analyses were followed in this review (13).

## Secondary Thalamic Injury

The degeneration of remote but functionally connected brain regions is originally termed “diaschisis” by Constantin von Monakow in the early twentieth century (14). By the 1990's, computed tomography (CT) and magnetic resonance imaging (MRI) detect thalamic atrophy in patients with cerebral infarction caused by an occlusion of the middle cerebral artery (MCAO) (3, 4). Concurrently, several groups describe secondary degeneration in the thalamus following rodent models of cortical lesion (15–17). Kataoka et al. first report ipsilesional thalamic injury following ischemic stroke in a rat model of MCAO (10). They observe decreased succinate dehydrogenase activity and massive silver staining of degenerated synaptic terminals in the ipsilesional thalamus at Day 5 post-stroke when compared with sham-operated rats. Their findings suggest a delayed ipsilesional thalamic degeneration after cortical ischemic stroke (10). In another rodent study, electron microscopic analysis shows that the cytolytic nerve cell degeneration is somatodendritic without central chromatolysis in the secondary thalamic injury at Day 7 post-stroke (18). Since then, this phenomenon of secondary injury has gradually attracted attention in the field of ischemic stroke. Increasing efforts have focused on understanding the pathological changes of secondary thalamic injury using cellular and molecular biology tools, and modern imaging techniques.

### Methods and Strategies to Identify Secondary Thalamic Injury

Secondary thalamic injury can be induced in most animal models of cortical stroke, such as transient (9) or permanent MCAO (6), photothrombotic models (19), middle cerebral artery (MCA) embolization model (20) and endothelin 1-induced stroke model. Traumatic injury to the cortex can also cause secondary thalamic injury (21). Depending on the site of initial cortical lesions and the connected cortico-thalamic projections, secondary injuries can develop in different sub-divisions of thalamus (7). For example, the primary somatosensory cortex (S1) predominantly projects to the ventral posterior medial nucleus (VPM) and the posterior medial nucleus (PoM) of thalamus (22). When a primary ischemic injury is generated in S1, we observe a secondary thalamic injury in VPM and PoM at Day 30 after stroke (9). In other studies, a primary injury in the motor cortex can lead to a secondary thalamic injury in thalamic posterior area and ventral posterolateral nuclei (VPL) area (7, 23). In a bilateral endothelin-1-induced infarct in the pre-frontal cortex, secondary degeneration is observed in dorsomedial nucleus of the thalamus and retrosplenial cortex (24).

Histological methods have been commonly used to detect neuronal changes and injuries in both primary and secondary injury sites after stroke, including Fink-Heimer silver staining (6), Nissl staining (5, 9), Fluoro-Jade staining (25), and immunostaining with neuron specific antibodies such as neuronal nuclei (NeuN) and microtubule-associated protein 2 (MAP2) (9, 26). Darkly stained, shrunken nuclei and atrophic perikaryal are presented by Fink-Heimer silver staining in the ipsilateral thalamic nuclei at Day 7 after cortical stroke (6). Degenerating neurons stained by Fluoro-Jade appear in the secondary thalamic injury site starting as early as Day 3 after focal ischemic stroke, peaking at Day 5 and decreasing by Day 16 (25). Nissl-stained brain sections show increased staining in the ipsilateral thalamus at one-month post-stroke. Significant pyknotic stained debris and loss of normal neuronal structure are present in the ipsilesional thalamus at Day 30 post-stroke in a MCAO model (Figures 1B,C) (9). Immunostaining of NeuN indicates neuronal loss in the degenerative thalamus, shown by the significantly reduced numbers of NeuN-positive neurons in the ipsilesional thalamus (9). With the progression of the secondary thalamic injury, severe shrinkage of the ipsilateral thalamus starts from 2 weeks up to several months after focal cortical ischemia detected by hematoxylin and eosin staining or Nissl staining (27, 28). In addition to changes in neuronal pathology, changes in neuronal function are also detected in affected thalamic nuclei. For example, the average firing rate of neurons located at secondary injury-affected VPL area is significantly reduced 1 day after MCAO but can be partially recovered at the later time points (29). Another study reports that primary somatosensory cortical injury induces long-term reduction in intrinsic excitability and evokes synaptic excitation of inhibitory reticular thalamic nucleus cells (30). Besides neuronal injuries, inflammatory responses, amyloid beta protein (Aβ) depositions, apoptosis, and blood-brain-barrier (BBB) breakdown are other pathological features that have been detected by histological methods in secondary thalamic injury. We will discuss these features in more details in the later sections.

More recently, modern imaging methods such as CT (3, 31), positron emission tomography (PET) (7, 32, 33) and MRI (4, 8, 34) have been used to dynamically track the changes in secondary thalamic injury *in vivo* (35). Kuhl et al. report metabolism dysfunction in the ipsilesional thalamus, with reduction in glucose utilization and perfusion by emission computed tomography (ECT) after cerebral stroke. Meanwhile no structural damage is detected by x-ray (XCT) imaging in the same region (31). In a focal cortical stroke rat model using microPET imaging, a 10% reduction in glucose metabolism in the ipsilateral thalamus is observed after initial cortical stroke at Day 1 post-stroke, but return to normal at Day 8 (36). Another PET tracer study using the amino acid, cis-4-18F-fluoro-D-proline (D-cis-18F-FPro) demonstrates that the uptake of D-cis-18F-FPro tracer begins as early as Day 3 post-stroke and is congruent with microglia/macrophage activation in the ipsilesional thalamus (positive immunostaining of cluster of differentiation 11b, CD11b and cluster of differentiation 68, CD68), suggesting a secondary thalamic injury after cortical infarct (7). A case study using PET imaging reports a patient with cerebral ischemic infarction in the territory of MCA exhibiting decreased ipsilesional thalamus blood flow at Week 3 post-stroke (37). MRI has also been broadly used to study secondary thalamic injury after stroke. Using MRI in a rat MCAO model, hyperintensities of T2-weighted signals transiently appear in the ipsilesional thalamus at Week 3 after cortical stroke but disappear at Week 7. This increased extracellular water content suggests the presence of edema in the ipsilesional thalamus (8). In the same study, T2^*^-weighted imaging shows intense hypo-intensity in the ipsilesional thalamus, beginning at Week 7 and lasting up to Week 24 after stroke. The MRI changes correspond with histological validation of reactive microglia/infiltrating macrophages and chronic accumulation of iron (8), indicating MRI as a potential tool to detect longitudinal changes in the secondary thalamic injury. Similarly, van Etten et al. report hypointense signals in ipsilesional thalamus on T2^*^-weighted MRI of unilateral ischemic stroke patients, suggesting accumulation of toxic iron in secondary thalamic injury (38). In patients with focal cortical infarction, a delayed shrinkage in the thalamus is detected by MRI scanned at late phase (31.6 ± 16.6 months after the initial stroke) (39). Another MRI study also reports similar reduction in thalamic volume in patients after acute MCA territory stroke (40). We have used diffusion tensor imaging (DTI) in our secondary thalamic injury study and demonstrated that decreased loss of fiber tract density is associated with decreased volume of secondary thalamic injury after stroke (9). Together these advanced imaging methods combined with traditional histological methods have advanced our understanding of the secondary thalamic injury after ischemic stroke.

### Secondary Thalamic Injury Is Associated With Functional Behavioral Deficits

The thalamus is an integrative hub for functional brain networks and is involved in multiple cognitive functions (41). Patients with focal thalamic lesions exhibit disruptions in the cortical functional network (41). Preservation of the thalamic circuitry is one of the major determinants for the quality of hand motor recovery following acute brain ischemia in the adult (42). Therefore, it is expected that secondary thalamic injury may affect brain function and impede stroke recovery. Clinical studies in stroke patients support this hypothesis. Patients with thalamic lesions after stroke reveal disrupted threshold detection to vibrotactile stimuli in the presence of a concurrent competing contralateral input (43). Santos and colleagues also demonstrate that secondary degeneration of thalamic nuclei via diaschisis can be associated with verticality misperception after stroke (44). In another study that evaluates patients at 3 months after stroke, the thalamic microstructural abnormalities detected by DTI imaging correlate with lower verbal fluency performance, suggesting the secondary abnormalities in thalamus are related to cognitive dysfunction (45). A prospective cohort study by Kuchcinski and colleagues demonstrate that the secondary thalamic alteration after focal cortical injury independently contributes to poor functional, cognitive and emotional outcome (46). However, the role of secondary thalamic injury in stroke outcome remains debatable. On acute ischemic stroke patients, thalamic hypoperfusion is detected in CT imaging but is not correlated with neurological deficits evaluated by modified Rankin Scale (mRS) scores (47). However, the thalamic hypoperfusion is insufficient to determine the secondary thalamic injury in this study. The authors also state that the long-term neurological outcome of thalamic changes is undetermined, since their clinical outcome is evaluated at Day 90 post-stroke which remains in the subacute phase after stroke.

In animal studies, some functional behavior tests have been used to evaluate how secondary thalamic injury influences stroke outcome. Improved long-term behavioral function coincides with reduced secondary thalamic injury after stroke (9, 48). Overall, it remains to be elucidated how secondary thalamic injury affects long-term behavioral function and stroke recovery.

## Pathological Changes in Secondary Thalamic Injury

Emerging studies have shown multiple pathological changes associated with the secondary thalamic degenerative injury after cortical ischemic stroke, including excitotoxicity (17, 30), apoptosis (27, 49), Aβ accumulation, BBB breakdown (50), and inflammatory responses (5, 25). We will discuss them in detail in the following sections.

### Excitotoxicity

In primary ischemic stroke, glutamate-mediated excitotoxicity contributes to the spread of damage after cerebral ischemia (51). Excess glutamate stimulation of the ionotropic N-methyl-D-aspartate (NMDARs) receptors leads to a massive influx of Ca^2+^ and unregulated intracellular signaling, such as nitric oxide pathway (postsynaptic density protein 95/nitric oxide synthases, PSD95/nNOS), calpain pathway and transcription-dependent death signaling, which are all known to cause cell death (51). Recently, studies have linked this excitotoxicity to the secondary thalamic injury after stroke. In a model of cortical ablation-induced secondary thalamic injury, astrogliosis and elevation of glutamate decarboxylase levels are shown in the connected ipsilesional thalamus (17). In the same study, similar observations is observed after an intracortical injection of kainic acid, suggesting that excitotoxicity can lead to secondary thalamic injury (17). Another study further emphasizes the importance of excitotoxicity in secondary thalamic injury, by demonstrating that interruption of the interactions between NMDAR and PSD95 after low oxygen post-conditioning treatment can reduce secondary thalamic injury (23). Although these studies suggest that excitotoxicity is associated with the secondary thalamic injury, the underlying mechanisms of glutamate-mediated secondary thalamic injury remain to be further elucidated.

### Apoptosis

A few studies have reported apoptosis in the ipsilesional thalamus after focal cortical ischemic stroke. In rat MCAO model, the number of terminal deoxynucleotidyl transferase dUTP nick end labeling (TUNEL)-positive cells is increased in the ipsilateral VPM at Day 7 and 28 after stroke (52, 53). Following the same timeline, mitochondrial transmembrane depolarization is detected with elevated ratio of cleaved caspase-3/caspase-3 and cleaved caspase-9/caspase-9, indicating increased caspase activation and apoptosis in the ipsilesional thalamus post-stroke (53). On the contrary, a study using mouse focal cortical ischemia model reports the lack of TUNEL-positive cells in the thalamic neuronal injured area between 6 h up to 90 days post-stroke, suggesting that the thalamic neuronal injury may not be apoptotic (54). In another study, mRNA of caspase-3 is increased at Day 7 after MCAO in the ventral thalamic nuclei, while no change is observed in the expression of the anti-apoptotic family members (B-cell lymphoma 2 associated X protein and B-cell lymphoma-extra-large protein) (55). These findings provide observational evidence of apoptotic changes in the secondary thalamic injury but are not necessarily causative.

### Tau and Aβ Accumulation

Tau and Aβ proteins are characteristic pathological features in neurodegenerative diseases such as Alzheimer's diseases (56). Interestingly, recent studies also link these proteins with secondary thalamic injury after stroke (52, 57). Phosphorylated tau proteins are increased in VPM neurons at Day 7 and Day 28 after cortical ischemic stroke in rats (52). The majority of the phosphorylated tau-positive neurons are also TUNEL-positive, suggesting that the hyperphosphorylated tau may be responsible for the secondary thalamic injury through apoptotic pathways (52). Aβ precursor protein (APP) and Aβ have also been reported in the secondary thalamic injuries. APP and Aβ are detected at the terminal zone of the deafferented axons in the thalamus at 1 week after transient MCAO in rats, and last at least up to 9 months (57). Furthermore, the increased APP and Aβ are correlated with increased β-amyloidogenic processing of APP and the imbalanced Aβ degrading enzyme levels (58). In addition, Aβ accumulation parallels the severity of secondary thalamic injury post-stroke. When the severity of secondary thalamic injury is exacerbated by chronic stress, increased Aβ accumulation is observed in the ipsilesional thalamus (59). Similarly, reduction of Aβ deposits is accompanied with alleviated secondary thalamic injury in hypertensive rats (60). All of these findings suggest a strong association between APP deposit and the secondary thalamic injury after stroke. Therefore, it is understandable that APP deposition has been used as a marker to assess axonal damage in the thalamus after stroke (61). However, there are contrasting views that report the lack of association between Aβ deposition and secondary thalamic injury. In cynomolgus monkeys with neuronal loss in the affected thalamus, there are no signs of Aβ deposits in the thalamus and no detectable significant changes of Aβ peptides in the cerebrospinal fluid or plasma levels after 12 months post-stroke (62). A clinical study also reports no correlation between Aβ aggregates and cerebrovascular lesions of various location, severity, and age (63, 64). Whether the association of Aβ deposition and secondary thalamic injury is rodent-specific, or that Aβ is transiently expressed in the secondary thalamic injury is unclear.

### BBB Breakdown

BBB is an amalgamation of the unique traits of brain endothelial cells. Developed and maintained by the neurovascular unit, the BBB creates a homeostatic clamp for the central nervous system (CNS); it ensures the rigorous regulation of molecules, ions, and cells between the blood and the brain (65, 66). Many neurological disorders are associated with BBB dysregulation, including Alzheimer's disease, epilepsy, multiple sclerosis, traumatic brain injury and stroke (65, 67). In the context of secondary thalamic injury after stroke, several studies have described changes in the BBB structure, permeability and the associated tight junction proteins. Ling and colleagues report a neovascularization phenomenon in the degenerative ipsilesional thalamus after a primary stroke in the somatosensory cortex (68). Angiogenesis is prevalent at Day 7 post-stroke in the degenerative thalamus using bromodeoxyuridine (BrdU) co-staining with laminin, a common blood vessel marker (68). In other studies, albumin staining in the ipsilesional thalamus has revealed changes in BBB permeability (50, 69). Increased albumin extravasation and decreased tight-junction protein expression, such as zona occludin 1 (ZO-1) and occludin are shown in the ipsilesional thalamus as early as 24 h after cortical stroke, and persist through Day 14 after cortical stroke (50, 69). Aberrant angiogenesis resulting in a loss of BBB integrity may contribute to inflammatory responses and injuries in the thalamus after cortical stroke. It is important to assess BBB dysregulation in the secondary thalamic injury as it relates to inflammatory responses and neurodegeneration (50, 69). There have been a few reports of BBB disruption in the ipsilesional thalamus in chronic post-stroke time points. Future studies should investigate comprehensive spatio-temporal characterization of the BBB leakage and its role in secondary injury development.

## Inflammatory Responses in Secondary Thalamic Injury

Inflammatory responses are one of the main pathogeneses during the development of secondary thalamic degeneration (9, 25, 70, 71). Cytokine tumor necrosis factor-α is upregulated in the ipsilesional thalamus as early as Day 1 after stroke (72). There are many studies report changes in resident glia (microglia, astrocytes and oligodendrocytes) in the remotely connected thalamus after stroke. More recently, peripheral immune cells have also been reported to participate in the secondary thalamic injury.

### Microglia

Microglia are the principal immune cells responding to the pathological changes at the primary injury site after ischemic stroke (73, 74). Accumulated data indicate that microglial activation is a key feature of the secondary thalamic injury in both rodents and humans (75, 76). One month after cortical ischemia, the number of microglial-like cells, as well as putative markers of microglial structural reorganization (ionized calcium binding adaptor molecule 1, Iba-1), phagocytosis (CD68), complement processing (CD11b), and antigen presentation (MHC-II) are all elevated in thalamic PoM and VPM in mice (76). In rats with focal cortical infarctions, CD11b positive staining are shown in the secondary thalamic degeneration from Day 8 to Day 28 post-stroke, suggesting microglial/macrophage activation (7). Microglia/macrophage activation in the ipsilesional thalamus occurs at a delayed time point compared to the activation in the primary cortical injury site. In a time course study of cortical stroke, microglia/macrophage activation markers (CD68 and Iba1) increase at Day 7, peak at Day 14 and 28, and persist up to Day 112 in the ipsilesional thalamus (77). We also observed microglia with dynamic morphologies and gene expression changes as the secondary thalamic injury develops (78). Besides histological staining, PET imaging has also been used to monitor microglial activation after stroke. In patients with cortical infarct, quantitative PET imaging using a marker of microglial/macrophage activation ([^11^C]PK1195P) show increased uptake in the ipsilesional thalamus between 2 months and 24 months after stroke onset, suggesting activation of microglial/macrophage in the thalamus (75). In permanent MCAO mice, *ex vivo* PET imaging also detects increased binding of translocator protein (TSPO) in the ipsilesional thalamus from Day 3 up to Week 3 post-stroke, suggesting microglia and astrocyte activation in thalamic injury (33). However, current strategies used for detecting microglial activation in secondary thalamic injury are with poor specificity. For example, TSPO is also presented in astrocyte and endothelial cells. The markers that have been used in secondary thalamic injury are incapable of differentiating between resident microglia and peripheral macrophages, as these cell populations derive from the same lineage and share many common markers. Future studies with specific markers that can distinguish microglia from other glia and macrophages are required for elucidating the role of microglia in secondary thalamic injury.

Recently, a spatio-temporal analysis study reports differential process extension response of microglia in the secondary thalamic injury than in the primary injury (79). As a highly conserved response to localized damage, microglia rapidly extend their fine processes toward the site of injury. These processes promptly respond to injury through binding of ATP released from the damaged cells to P_2_Y_12_ receptors expressed on microglia. In Kluge and colleagues' study, laser damage is applied to the incubated brain sections as external stimulus, and the microglia/macrophages are labeled with GFP in Cx3CR1^GFP/WT^ mice (79). Microglia/macrophages in the degenerative thalamus lose the process extension feature upon laser damage but show an increase in the phagocytic function during the late phase after photothrombotic cortical stroke. These features are distinct from the microglia in the primary injury site, where microglia retain their ability of directed process extension after laser damage (79, 80). A follow-up study suggests the failed microglial responses to laser damage in the secondary injured thalamus is associated with P_2_Y_12_ receptor distribution changes and a disruption of ATP gradient required for sensing and detection (79).

All of these studies reveal a tight link between microglia activation and the progression of secondary thalamic injury after stroke. Some studies have attempted to explore the roles of microglia in the secondary thalamic injury. For example, Justicia et al. show that the expression of heme oxygenase (HO-1), a heat shock protein, is increased in cells co-expressed with microglial markers in the ipsilesional thalamus 3 weeks after stroke. The induction of HO-1 can be interpreted as an index of microglial and macrophage stress response to injury (8). Another study shows increased engulfed NeuN pixels by microglia in the ipsilesional thalamus at Day 56 after stroke (79), suggesting microglia may play a role in phagocytosis after secondary thalamic injury. However, the role of microglia in the secondary thalamic injury and their effects on long-term behavioral function are still unclear; whether microglia/macrophages are causes or consequences of the secondary thalamic injury are unknown.

### Astrocytes

**Figure 3:**
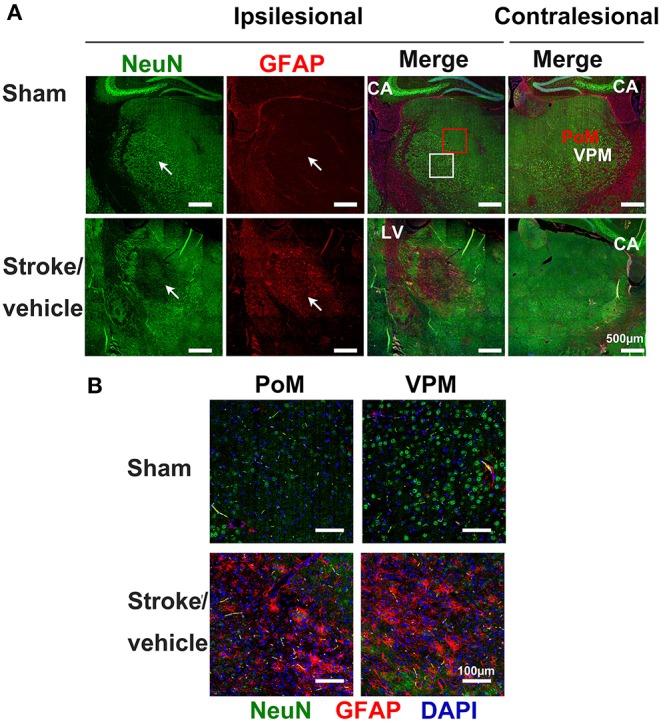
Inflammatory responses in the secondary thalamic injury after primary cortical stroke. **(A)** Immunostaining shows neuronal loss and reactive astrocytes clustered in the ipsilesional thalamic area (indicated by arrows) at 1 month post-stroke. Note that sham animals exhibit healthy neurons in the thalamus and reactive astrocytes were not detected. Anti-NeuN stains neurons, anti-GFAP stains astrocytes, and DAPI stains nuclei. Scale bar = 500 μm. CA, cornu ammonis; LV, lateral ventricle. A red square marks the posteromedial complex (PoM) and a white square marks the ventral posteromedial nucleus (VPM) area on thalamus. **(B)** Enlarged images from PoM and VPM from ipsilesional thalamus. Scale bar = 100 μm. Adapted from Cao et al. (9), under the CC BY license.

Astrocytes are the most abundant glial cells in the brain that control many functional aspects of the CNS in both health and disease. Astrocytes respond to changes and injuries initiated by ischemic stroke. Activated astrocytes are characterized by hypertrophy of their cellular processes with altered gene expression, such as upregulated astrocyte-specific cytoskeletal protein, glial fibrillary acidic protein (GFAP) (Figure 3). In the primary stroke injury site, reactive astrocytes play both beneficial and deleterious roles at different stages after stroke; this has been well-summarized by previous reviews (81, 82). Astrocytes are strongly activated in the secondary thalamic injury area in multiple ischemic cortical stroke models (9, 83–86). In a photothrombotic occlusion of somatosensory/motor cortex mouse model, the intensity of GFAP expression showed 63.75% increase in ipsilesional thalamus at Day 28 post-stroke when compared to sham animals, suggesting strong astrogliosis in the secondary thalamic injury (86). Similarly, GFAP mRNA and immunoreactivity are significantly increased in the ipsilesional thalamus after focal cortical stroke in rat (84). In a secondary thalamic injury induced by cortical excitotoxic lesion, astroglia hypertrophy, and increased GFAP expression transiently appear in the thalamus, whereas the astrocyte changes can last up to 30 days in cortex after injection of N-methyl-d-aspartate (87). It is unclear if this differential profile of astrocyte activation between the primary cortical injury and secondary thalamic injury also exists after ischemic stroke. Similar to microglia, the role of astrogliosis in secondary thalamic injury is still uncertain. It is noted in an aging study that the severity of the secondary thalamic injury is exacerbated in aged mice, despite similar levels of microglia and astrocyte activation between young and aged stroke mice (88).

### Oligodendrocytes

Oligodendrocytes are key glial cells responsible for producing myelin sheaths that wrap around neuronal axons in CNS. Myelin is essential to propagate action potentials rapidly and to support axons metabolically (89). Oligodendrocytes have been shown to crosstalk with microglia and astrocytes to mediate demyelination and remyelination (90), which are important processes in stroke recovery. Excitotoxicity, oxidative stress, and inflammation cause oligodendrocyte cell death and demyelination in primary ischemic stroke (91–93). At present, very few studies report the involvement of oligodendrocytes and myelination in secondary thalamic degeneration. Myelin basic protein (MBP) is detected as early as Day 14 in the ipsilesional thalamus after cortical stroke (94), suggesting that changes in myelination (demyelination and/or remyelination) occurs in the secondary thalamic injury. In another study, Wang and colleagues show that the oligodendrocyte marker RIP (Rip-antigen), is significantly increased in the ipsilateral VPM in the first week after cortical injury, suggesting activation of oligodendrocytes in the secondary thalamic injury. Also, Nogo-A persistently increases in RIP labeled oligodendrocytes through 4 weeks (61). However, it is unknown if oligodendrocytes release Nogo-A as a response to the secondary thalamic injury. Currently, what is known about the involvement of oligodendrocytes in secondary thalamic injury is very limited; more studies are needed in this field given the importance of oligodendrocytes and their interaction with other types of glia in myelination.

### Peripheral Immune Cells

Peripheral infiltrating immune cells are pivotal in the inflammatory responses in primary infarct area after stroke (95–98). At present, only one study reports the involvement of peripheral infiltrating immune cells in the secondary thalamic injury. In a photothrombotic cortical stroke model, flow cytometry analysis indicates that CD4^+^ and CD8^+^ T cells are significantly increased when compared to the contralesional thalamus at Day 14 after stroke; conversely no changes are detected in B cells, neutrophils, and monocytes (19). Positive immunostaining of CD3^+^ (a common component of all T cells) in the ipsilesional thalamus also supports this finding (19). However, the authors only test a single time point, Day 14 post-stroke and which is relatively early. Further studies are needed to confirm the significance of peripheral immune cells in the secondary thalamic injury.

## Interrogation Studies in Secondary Thalamic Injury After Stroke

As the secondary thalamic injury progressively and chronically develops after stroke, this delayed characteristic provides an optimal and longer time window for treatment. Recent efforts have been made to interrogate the role of various molecules and processes in the development of secondary thalamic injury after ischemic stroke. Some studies have shown that interventions to prevent secondary thalamic injury may be beneficial for recovery of function after stroke. Below is a summary of studies that investigate the role of secondary thalamic injury through manipulating specific molecules involved in inflammatory responses, autophagy, Aβ deposition, and neuronal apoptosis (5, 26, 50, 59, 60, 99–101).

Osteopontin (OPN) is a major secretory product of activated macrophages known to have pro-inflammatory and anti-inflammatory effects. It also downregulates iNOS expression to reduce inflammation (5). OPN-knockout mice exhibit a robust increase in retrograde degeneration in the thalamus without changes in cortical infarct size (5). Interestingly, the observed increase in thalamic neurodegeneration at Day 14 post-stroke is accompanied by a robust surge in microglia activation as well as increased mRNA expression of several pro-inflammatory genes (5). Another intervention to manipulate secondary thalamic injury is to target the autophagic processes (100, 101). A shRNA-induced knockdown of beclin1, a regulator of autophagy, reduces autophagy, astro/micro-gliosis, and neuronal cell death in the ipsilesional thalamus (100). In another study, cathepsin-B (CathB), a lysosomal marker that increases as a result of autophagosome degradation, has been shown to progressively increase in the thalamus through 28 days after stroke, and its expression corresponds with the increased astroglia/microglia activation and neuronal cell loss (101). Pharmacological inhibition of CathB reduces inflammation and prevents neuronal injury and apoptosis in the ipsilesional thalamus (101), indicating the importance of this gene and the autophagy process in secondary thalamic injury after stroke. Other treatments such as the non-selective calcium channel blocker bepridil (102) and antioxidant ebselen (103) also decrease secondary thalamic injury in different cortical stroke models.

A few studies have investigated the intervention of secondary thalamic injury on long-term behavioral functions after stroke. Cerebrolysin treatment reduces Aβ deposition, apoptosis and autophagy in the thalamus and improves functional recovery after cortical infarction (26). Netrin-1 ameliorates the impairment of BBB in the ipsilesional thalamus by promoting tight junction function and endothelial survival at Day 14 post-stroke (50). Meanwhile, an improved neurological function is assessed at the same time point (50). Previously, using pharmacologically induced hypothermia, we observe reduced neuronal loss and less astrogliosis in the ipsilesional thalamus through 28 days after stroke (9). This reduced secondary thalamic injury and alleviated neuroinflammatory responses is accompanied by long-term behavioral functional recovery (9), although the improved behavioral function may result in alleviation of both primary cortical injury and secondary thalamic injury. Another study by Anttila et al. administrated cerebral dopamine neurotrophic factor (CDNF) and mesencephalic astrocyte-derived neurotrophic factor (MANF) directly into the thalamus at Day 7 post-stroke (104). Although CDNF and MANF treatment did not affect the thalamic neuronal loss or phagocytosis in the thalamus, both treatments promote the functional recovery (104). Future studies that selectively target the secondary thalamic injury will elucidate the relationship between secondary thalamic injury and long-term stroke outcomes, and contribute to developing novel strategies to improve recovery after stroke.

## Future Perspectives

Stroke is currently being viewed as a disorder of brain connectivity. A damaged stroke area can cause network-wide deficits in areas adjacent to or remotely connected to the infarct. In particular, the connected thalamus undergoes degeneration after a cortical stroke. This is likely due to the lack of neuronal network activity between the cortex and the connected thalamus. Our group has previously demonstrated that post-stroke optogenetic neuronal stimulations can directly increase the neuronal activity of the primary motor cortex (iM1) and its connected regions, and repeated iM1 neuronal stimulations increased expression of neurotrophins, enhanced cerebral blood flow and motor function after stroke (105). Optogenetic approaches can be useful for studying secondary thalamic degeneration after stroke. A study by Brown's research group demonstrates that selective optogenetic stimulations of the thalamo-cortico circuit can enhance recovery post-stroke (106). As the primary stroke site occurs in the cortex, it is likely that restoring activity in the cortico-thalamic circuit could provide substantial benefits in stroke outcome. It is expected that selective optogenetic stimulation of the cortico-thalamic circuit can re-introduce neuronal activity in the cortico-thalamic network, which potentially may reduce secondary degenerative injury and attenuate inflammatory responses in the ipsilesional thalamus.

**Figure 4:**
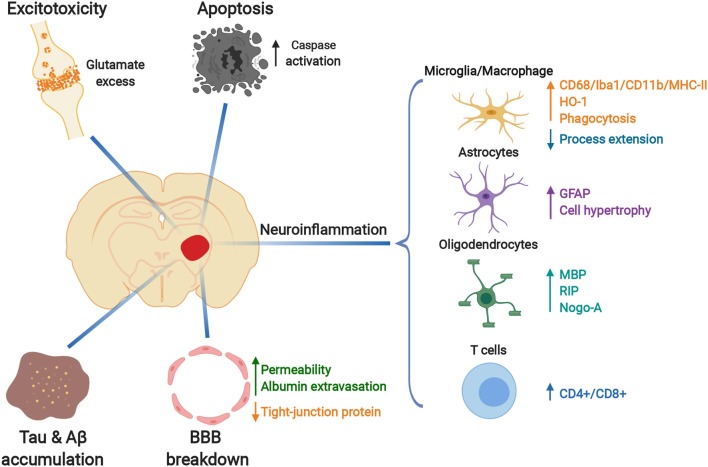
Pathological changes in the secondary thalamic injury after primary cortical stroke. Glutamate-related excitotoxicity, increased caspase activation and apoptosis, Tau and Aβ protein accumulation, blood-brain-barrier (BBB) breakdown and activated inflammatory responses occur in the ipsilesional thalamus after cortical ischemia. Resident glia (microglia, astrocytes, and oligodendrocytes) are activated during the development of secondary thalamic injury, with dynamic morphological and molecular changes. Peripheral T cells infiltrate into the ipsilesional thalamus. Created with BioRender (https://biorender.com).

In this review, we have discussed emerging studies characterizing the pathological changes in the secondary degenerative thalamus after stroke, including excitotoxicity, apoptosis, Aβ accumulation, BBB breakdown and inflammatory responses, as well as multiple cell types involved in these processes (Figure 4). However, the underlying cellular and molecular mediators driving the development of secondary thalamic degenerative injury remain to be elucidated. Future studies using high throughput technologies such as RNA sequencing of specific sorted cell types and single cell RNAseq can provide important insights into key molecular and cellular mediators. Ultimately, understanding the causative pathological mechanisms of secondary injury after stroke can reveal potential drug targets for enhancing recovery. Furthermore, the knowledge gained from secondary degenerative injury studies will also benefit the investigation of other neurological/neurodegenerative diseases.

## Author Contributions

ZC, SH, MC, TB, and GS contributed to the concept and design of the manuscript. ZC and SH performed literature review for the manuscript. ZC, SH, and MC wrote the manuscript. ZC and MC made the figures. TB and GS contributed in revising the manuscript. All authors contributed to revising the manuscript, reading, and approving the submitted version.

### Conflict of Interest

GS is a consultant for Qool Therapeutics, Peter Lazic US, Inc., NeuroSave, SanBio, Zeiss and Surgical Theater. The remaining authors declare that the research was conducted in the absence of any commercial or financial relationships that could be construed as a potential conflict of interest.
